# Long-Range Interactions Restrict Water Transport in Pyrophyllite Interlayers

**DOI:** 10.1038/srep25278

**Published:** 2016-04-27

**Authors:** Piotr Zarzycki, Benjamin Gilbert

**Affiliations:** 1 Institute of Physical Chemistry, Polish Academy of Sciences, Warsaw, Poland; 2 Lawrence Berkeley National Laboratory, Berkeley, CA 94720, United States

## Abstract

Water diffusion within smectite clay interlayers is reduced by confinement and hence is highly determined by the interlayer spacings that are adopted during swelling. However, a molecular understanding of the short- and long-range forces governing interlayer water structure and dynamics is lacking. Using molecular dynamics simulations of water intercalated between pyrophyllite (smectite prototype) layers we provide a detailed picture of the variation of interlayered water mobility accompanying smectite expansion. Subtle changes in hydrogen bond network structure cause significant changes in water mobility that is greater for stable hydration states and reduced for intermediate separations. By studying pyrophyllite with and without external water we reveal that long-range electrostatic forces apply a restraining effect upon interlayer water mobility. Our findings are relevant for broad range of confining nanostructures with walls thin enough to permit long-range interactions that could affect the mobility of confined solvent molecules and solute species.

Clays are very abundant layered minerals in the Earth’s crust. They influence soil permeability and chemistry and have important technological roles such as substrates for catalysis^1^. Smectites are 2:1 phyllosilicates (see Fig. 1a) that are a subject of enduring interest due to their ability to exchange, retard and sequester ions, which makes them promising components of geologic and engineered repositories for nuclear waste and CO_2_^1^,^2^.

The sorption and transport properties of water in smectite system are highly correlated with its hydration and swelling behavior^1^,^2^,^3^. In a regime of low water activity or high confining pressure, the expansion of smectites proceeds by an increase of layer separation in a stepwise fashion. In this regime, only discrete separations that correspond to an integer number of water layers (*i.e*., 1W, 2W, 3W, …) are observed experimentally^1^,^2^,^4^,^5^,^6^. These stable hydration states are separated by energy barriers associated with the energetic cost of changing the pattern of confined water hydrogen bonding^7^,^8^. The transition between these stable hydration states (*e.g.*, 1–2W, 2–3W, 3–4W, …) is a first-order phase transition^2^,^4^,^9^, and intermediate states are rarely observed^10^ even though the observation of swelling hysteresis^3^,^9^ suggests a continuous transition.

The dynamics of water molecules confined between smectite layers differ from bulk water. Both simulations and experiments^6^,^9^,^11^,^12^,^13^ report a lower self-diffusion coefficient of trapped water as compared to the bulk water, which approaches the macroscopic value as clay hydration (swelling) progresses. It has been shown that interlayer water mobility is determined by several short-range interactions including intermolecular hydrogen bonding^14^,^15^ and water-mineral interactions that are significant for highly confined fluids^16^,^17^. However, we currently lack a complete description of the relationship between smectite swelling and interlayer water mobility. Here we report the results of simulations that predict that long-range electrostatic interactions exert an unexpected influence upon interlayer water mobility.

We used molecular dynamics (MD) simulation to predict the mobility of water molecules within two uncharged smectite layers (i.e., pyrophyllite) as a function of layer separation. The ideal pyrophyllite structure does not contain Si or Al vacancies or metal substitutions that confer a permanent charge to the layer and thus do not require the presence of interlayer cations for charge compensation. Figure 2 summarizes the results of simulations that were performed at a large number of fixed interlayer separations from 6.5–30 Å.

As shown by the plots of interlayer water density profiles (Fig. 2c), well-defined 1, 2 and 3-layer hydration states develop at layer distances that agree with experimental observations of low-charge smectite^6^,^9^,^11^,^12^,^13^. Although these stable hydration states exhibit the most structured water perpendicular to the layers, the plot of the mean lateral diffusion rates for each configuration (Fig. 2b) reveals that these states also have the highest water mobility parallel to the layers. The mobility of the interlayered water oscillates as the layer separation continuously increases with local maxima in mobility for the stable hydration states and minima for the transition states.

Because prior work has suggested that transient states are characterized by disruption of the interlayer hydrogen bond (HB) network, we analysed the water HB pattern using a simple geometric criterion^14^,^18^ (see Supporting Information). Figure 2d shows lateral profiles of the total HB number, and the HB acceptor and donor numbers, for layer separations corresponding to stable or transition hydration states. The trend with separation is reported in Figure S7. The HB number for interlayer water molecules increases steadily but does not exhibit similar oscillations as water mobility.

The profiles of HB acceptors and donors demonstrate that the water molecules are oriented differently inside the interlayer as expansion progresses. At small separations, the HB-acceptors (water O atoms) are located closer to the clay-plates than HB-donors (water H atoms), which in turn populate the center of the interlayer (*e.g.*, 1W, 1–2W; Fig. 2d). As the hydration progresses, both acceptors and donors predominantly occupy the center of interlayer (2W, 2–3W; Fig. 2d), but as expansion increases further the HB-donors start to dominate in the vicinity of the pyrophyllite layers (3W, 3–4W; Fig. 2d). These represent significant reorientations of the interlayer molecules, which strongly influenced water mobility.

We note that this pyrophyllite model predicts different HB conformations than charged smectites and interlayer cations. For example, the presence of interlayer cations is predicted to cause the surface water molecules to reorientate^7^,^8^. However, the present model serves to investigate short- and long-range interactions affecting water mobility.

Although short-range interactions between interlayer water molecules did not simply explain trends in mobility, our simulations predicted that waters situated outside of the interlayer region exerted a surprising long-range influence on the mobility of water molecules within. As shown in Fig 2b, there is a significant decrease in predicted interlayer mobility when water is present in the external region, compared to when this region contains a vacuum. This prediction is striking because external water had no discernable effect on the time-averaged profiles of interlayer water (Fig. 3) or on the HB patterns.

We repeated these simulations for external water that was fixed at a number of configurations selected from a 5-ns fully dynamic simulation (see Supporting Materials). Static external water exerted a very similar effect of reducing interlayer water mobility (Figure S3). These comparisons implicate water-water interactions that extended farther than the 12-Å distance between water molecules on each side of a pyrophyllite layer (cf. Figure S8). As shown in Figure S9, the electrostatic forces must be responsible because the van der Waals forces described by a Lennard-Jones potential are negligible beyond ~6 Å. Although our fully atomistic simulations do not explicitly consider the shielding of electrostatic forces by dielectric materials such as the smectite layer, Figure S9 also shows that the interactions remain significant for reasonable values of the relative permittivity of water (~70) or clay (5–40).

Although there have been many studies showing the preference for the fully developed hydration layers^4^,^5^,^6^,^7^,^10^,^11^,^13^,^19^,^20^, this is believed to be the first study showing that the formation of stable and transition states affects the mobility of water confined between uncharged smectite layers. Moreover, our simulations predict that external aqueous solutions can restrict the mobility of water confined within thin layers. We anticipate the finding of an ‘electrofriction’ has relevance to aqueous solutions confined in numerous materials including inorganic nanopores and nanotubes, zeolites and other nanoporous structures^17^. Long-range interactions across confining smectite layers may also play an influential role in determining the mobility of intercalated ions and other species, and will be the topic of future work.

## Computational Methods

Molecular dynamics simulations were carried out initially in the NPT ensemble at the ambient temperature and pressure for 5 ns (Berendsen barostat-thermostat, relaxation constants equal 0.5 ps; temperature T = 25 °C, pressure p = 1atm)^21^,^22^, next in the NVT ensemble (Hoover thermostat, thermostat relaxation time τ = 0.5 ps, T = 25 °C)^21^,^22^ for another 10 ns; the trajectory of the last 5 ns was analysed with respect to the water mobility, atomic densities and hydrogen-bonding. Simulations were carried out using the DL_POLY 4.1 package^23^. We used the Smooth Particle Mesh Ewald for the electrostatic interactions (k-space evaluated every 0.001 ps, Ewald convergence equals 0.32 Å^−1^). The cutoffs for the Lennard-Jones interactions and real space in Ewald summation were set to 20 Å. The structure of pyrophyllite^24^ – a non-swelling and uncharged clay – was used to construct the prototypical clay slabs (flexible lattices in our simulations). The charge neutral, non-swelling smectite was chosen to avoid any interference coming from the ions (i.e., isomorphically substituted and/or counterions) on the dynamics of interlayered water. The interactions were described using the CLAYFF force field^25^ and the SPC/E water model^26^. We used the periodic boundary conditions, which do not affect the diffusion coefficient of interlayered species^19^.

Diffusion coefficient was calculated from the mean square displacement via the Einstein relation^21^,^22^. The water hydrogen bonding was analysed using the geometric criterion (i.e., intermolecular O-O distance <3.5 Å angle between O-O axis and one of the H-O bonds is lower than 30°)^14^,^18^.

## Additional Information

**How to cite this article**: Zarzycki, P. and Gilbert, B. Long-Range Interactions Restrict Water Transport in Pyrophyllite Interlayers. *Sci. Rep.* **6**, 25278; doi: 10.1038/srep25278 (2016).

## Supplementary Material

Supplementary Information

## Figures and Tables

**Figure 1:**
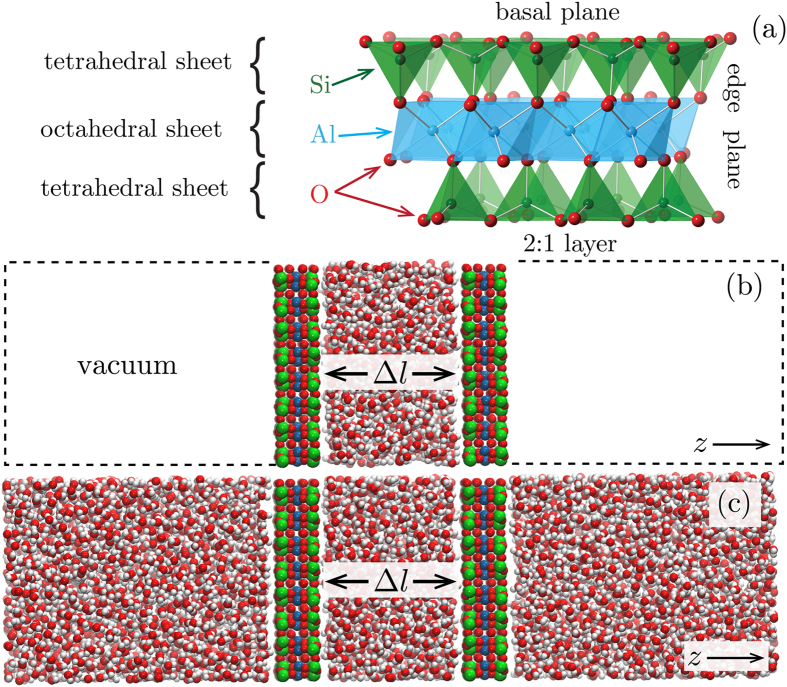
(**a**) The structure of pyrophyllite clays (charge neutral analog of smectites). Two tetrahedral sheets of silica sandwich an octahedral sheet of alumina. **(b**,**c)** The simulation cells. We used two types of simulation models to understand the propagation of structural and dynamic correlations across the clay plates: with water present only between the clay plates (**b**) and with water also outside the plates interlayer (**c**).

**Figure 2:**
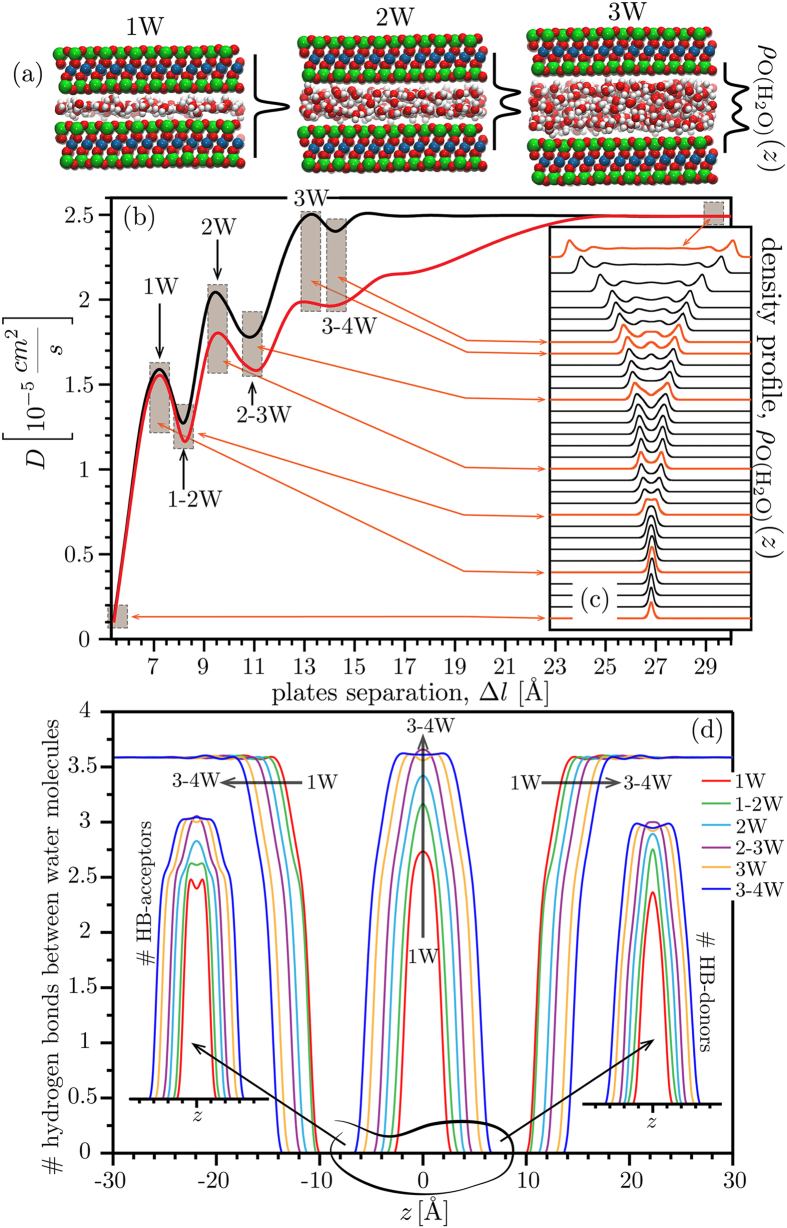
Variation in the interlayer water diffusion coefficient with uncharged smectite (i.e., pyrophyllite) layer separation for two simulation models (**b**): water inside the pyrophyllite layers and vacuum outside (**black**) and water both between and outside of the layers (**red**). The brown shaded boxes in panel b identify the stable hydration states (1W, 2W, 3W as visualized in (**a**)) or the intermediate states (1–2W, 2–3W, 3–4W). The corresponding oxygen density profiles in (**c**) are indicated by arrows. Panel d shows the calculated water-water hydrogen bonding profiles for key separations (i.e., 1W, 1–2W, 2W, *etc*).

**Figure 3:**
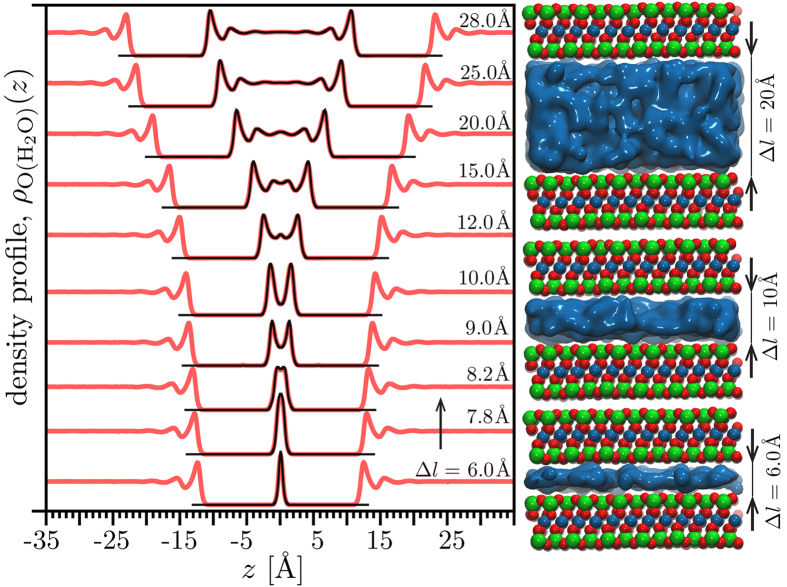
Water oxygen density profiles for the model with water only between plates (black) and also with water present outside the plates (red) for a varying clay-plates spacing. The interlayer profiles are almost indistinguishable.
