# Supplementary material for: Long-Range Interactions Restrict Water Transport in Pyrophyllite Interlayers
Source: Sci Rep. 2016 Apr 27;6:25278. doi: 10.1038/srep25278 (PMC4846866; doi:10.1038/srep25278)
Supplement: Supplementary Information [file srep25278-s1.pdf]

Supporting Information for: Long-Range Correlations Restrict Water Transport in Pyrophyllite Interlayers

Piotr Zarzycki,*† Benjamin Gilbert‡

Atomic densities

The atomic density profiles $\rho(z)$ are calculated similarly to our previous studies by using the following formula [3] :

$$\rho_k(z) = \frac{1}{N_{\text{frames}}} \sum_{j}^{N_{\text{frames}}} \sum_{i}^{N_k} \delta(z_{k,ij} - z) \tag{S1}$$

where $z_{k,ij}$ is z-coordinate of atom i of type k in the configuration j (simulation frame).

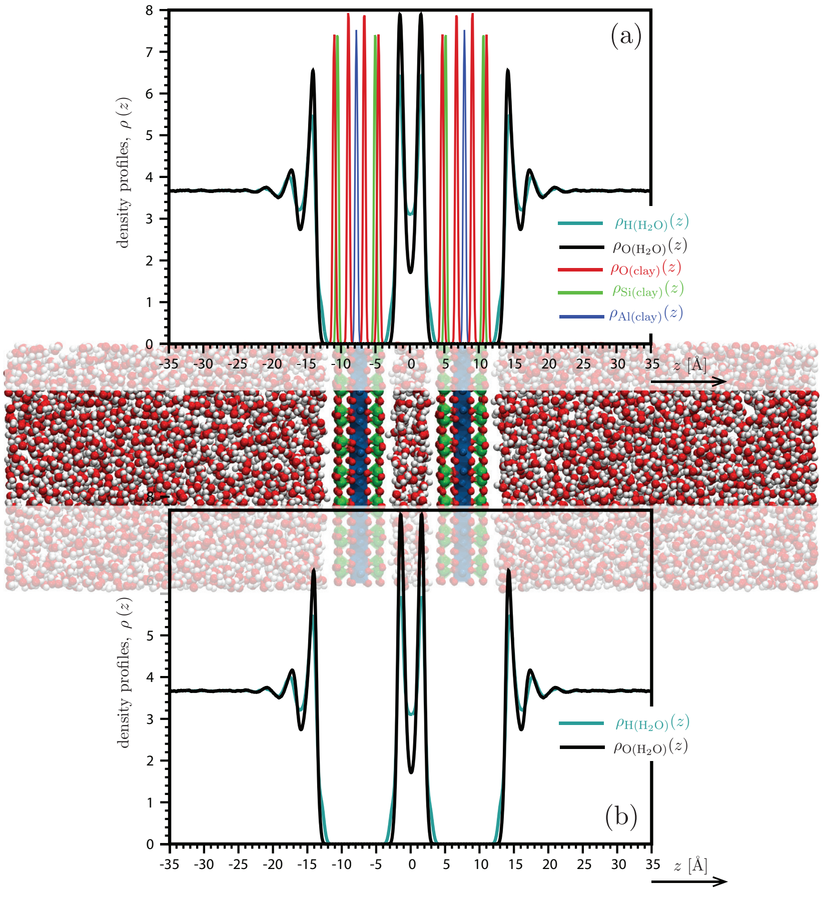

Figure S1: Atomic density profiles for all species in the computational cell (a) and for only water oxygen and hydrogen atoms (b). Illustrated for the clay plate separation equals 9.6 Å. For clarity of presentation, we rescaled the atomic densities (clay atoms, $\rho_{\text{H(H}_2\text{O)}}$) to fit to the range of $\rho_{\text{O(H}_2\text{O)}}$.

*Prof. Piotr Zarzycki, Institute of Physical Chemistry, Polish Academy of Sciences, Warsaw, Poland
†e-mail: zarzycki.piotrek@gmail.com
‡Prof. Benjamin Gilbert, Lawrence Berkeley National Laboratory, Berkeley, CA

Static external water ensemble affects the interlayer water mobility

The dynamics of the interlayer water is affected by the presence of the water ensemble outside the clay plates (see **main report**). However, it remains unclear if this is a dynamic (across-plate coupling in the molecular motion) or static effect (i.e., restraining electrostatic field across the clay plate). To understand the origin of restrained interlayer water dynamics we carried out the additional simulations with all water molecules external to the interlayer region frozen in an energetically optimal configuration.

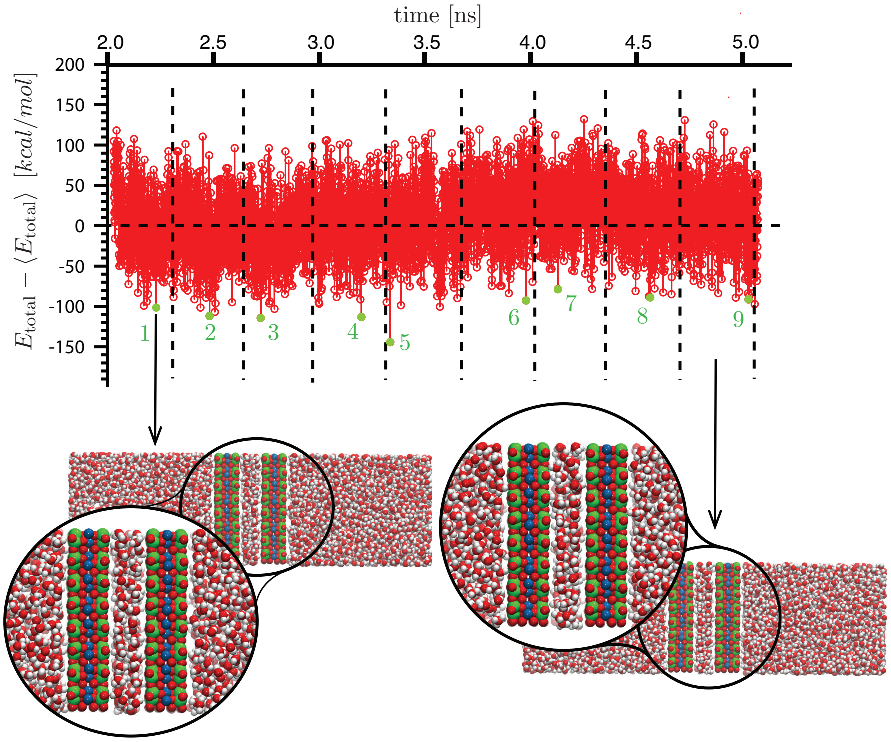

Figure S2: Configurations selecting for the sequence of simulations with the water outside the clay plates dynamicaly frozen (constrained xyz-position). The 3-ns part of the trajectory is divided into 10 windows (300 ps each), the lowest total energy configuration within the considered window is selected as an input (i.e., one with the locally highest Boltzmann weight).

Methodology: We reanalyzed all trajectories obtained from the unrestrained molecular dynamics of system with water between clay plates and outside. We calculated the microstate probability (i.e., Boltzmann weight factor $\exp(-E/k_B T)$) for each configuration. Next, we divided the last 3 ns of trajectory into timeframe windows (300 ps each, see Fig. S2), identified the lowest energy configuration within each window (i.e., microstate with a highest probability of occurring) and used is an input for the additional modeling with outside-water dynamically frozen (see Fig. S2). Finally, we compare the diffusion coefficient for the interlayer water with the restrained (control experiment) and unrestrained outside water molecules.

Results: The frame-averaged interlayer diffusion coefficient for restrained outside water modeling are shown in Fig. S4 Surprisingly, the constraints imposed on the dynamics of the water outside the clay plates have very little effect on the interlayer water mobility. We interpret this as a manifestation of structure to dynamics coupling, that is the outside-plate water ensemble exerts the long-range time-independent electrostatic friction on the water confined between clay plates.

In Fig. S4 we show the variation in the interlayer water diffusion coefficient among selected configurations for each plate separation in a control experiments (illustrated for two cases in Fig. S5). This variation, presented as the error bar (Fig. S4), decreases with increasing amount of the interlayer water (i.e., with layering). We believe that decreasing variation originates from the improved mean-square displacement statistics and it does not seem to have any physical importance.

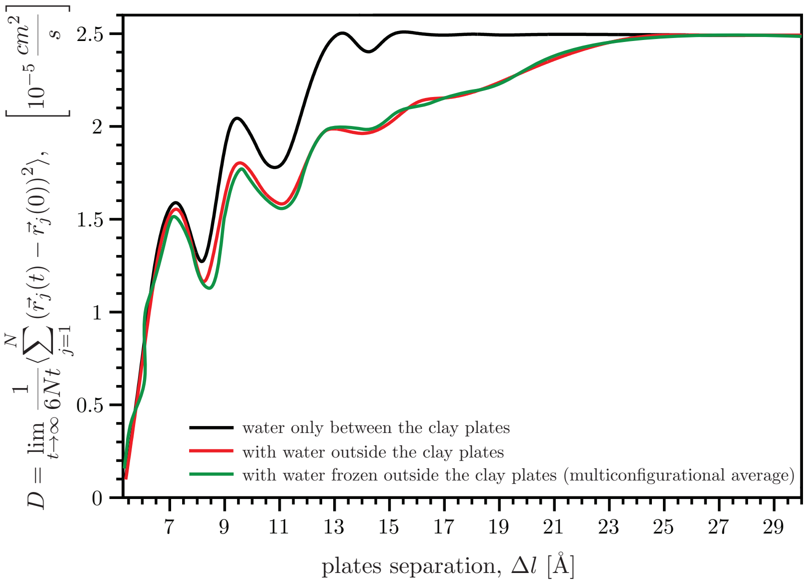

Figure S3: Interlayer water diffusion coefficient for the model with water only between clay plates (**black**), with the dynamically unrestricted water ensemble outside clay plates (**red**) and with the frozen water outside the clay plates – dynamically constrained (**green**).

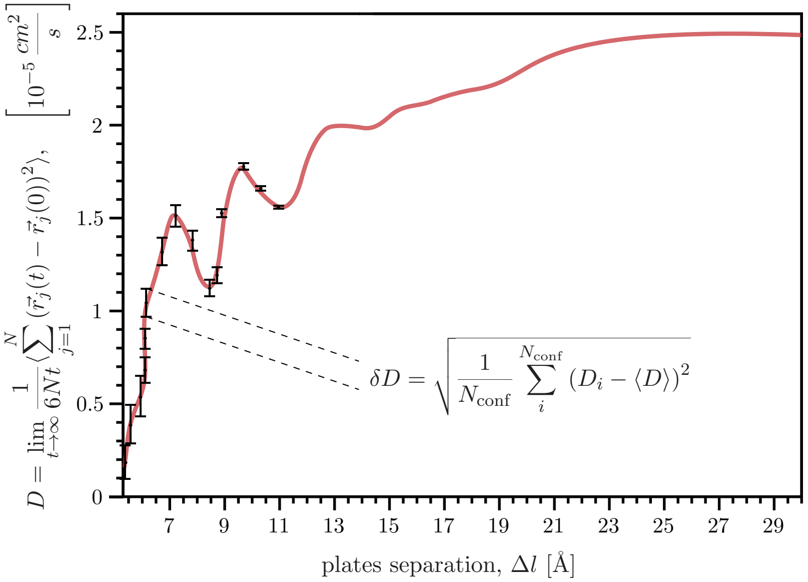

Figure S4: Variation in the diffusion coefficient for the interlayer water among the selected frozen water configurations (constrained dynamics of the outside-plates water). The variation (shown as the error bar) decreases with the increasing amount of the interlayer water, that is with improving the statistics of recorded mean-square displacement of interlayer water molecules. For plane separation larger than 11 angstroms the variation in the calculated interlayer diffusion coefficients is very small and thus not shown.

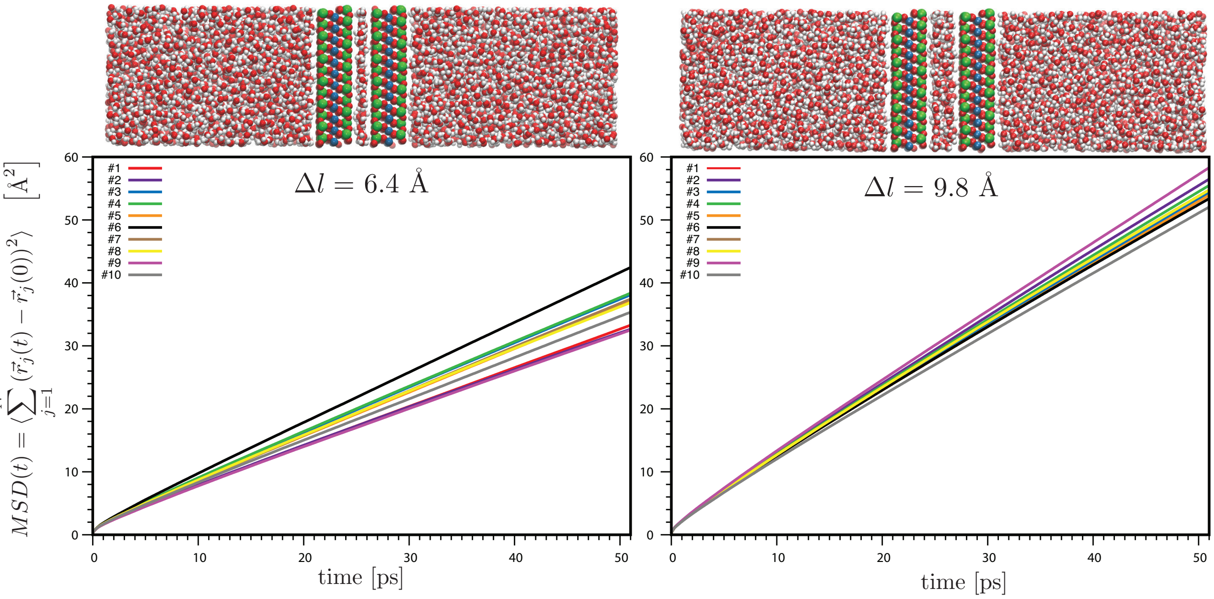

Figure S5: Variation in the mean square displacement (MSD) of the interlayer water among 10 selected configurations of the outside water, which was kept frozen in molecular dynamics simulations. MSD is shown for two clay plate separation ($\Delta l = 6.4$ Å and $\Delta l = 9.8$ Å). Molecular representation of the simulation cell is shown on above the MSD plots.

Table S1: Hydrogen bonding statistics for the key clay-separations corresponding to the fully developed water layers (1W, 2W, 3W) and intermediate spacing corresponding to the oxygen density peak splitting/restricted water mobility (1W→2W, 2W→3W, 3W→4W).

Configuration (see Fig. 2 in the manuscript)	Average number of hydrogen bonds $\langle N_{\mathrm{H-bonds}} \rangle$	Average number of H-bond donors $\langle N_{\mathrm{H-bonds(donors)}} \rangle$	Average number of H-bond acceptors $\langle N_{\mathrm{H-bonds(acceptors)}} \rangle$
water only between the clay-plates; vacuum outside			
1W	1.82	0.80	0.98
1W→2W (1-2W)	1.99	0.92	1.07
2W	2.32	1.12	1.21
2W→3W (2-3W)	2.52	1.21	1.30
3W	2.71	1.32	1.39
3W→4W (3-4W)	2.84	1.39	1.45
water between and outside the clay-plates			
1W	1.70	0.77	0.93
1W→2W (1-2W)	1.86	0.86	1.04
2W	2.22	1.09	1.19
2W→3W (2-3W)	2.48	1.21	1.28
3W	2.64	1.31	1.38
3W→4W (3-4W)	2.76	1.34	1.41

Hydrogen bonding

We analyze the hydrogen bonding pattern between water molecules using the simple geometric criterium. Two water molecules are considered as hydrogen-bonded if the intramolecular oxygen-oxygen distance ($r_{\mathrm{O-O}}$) is lower than 3.5 Å and the angle between oxygen-oxygen axis and one of oxygen-hydrogen bonds (ϕ) is bellow 30° (see Fig. S6).[1, 2]

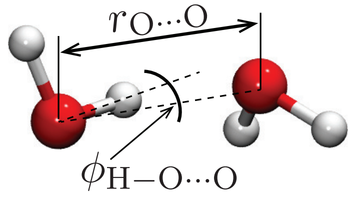

Figure S6: Geometric criterion for the hydrogen bonding between water molecules: intra-molecular oxygen-oxygen distance $r_{\mathrm{O\cdots O}} < 3.5$ Å, and angle between $\mathrm{O\cdots O}$ axis and one of the H-O bonds $\phi_{\mathrm{H-O\cdots O}} < 30°$.[1, 2]

The average number of hydrogen bonds (HB), HB-donors and HB-acceptors for the confined water and their values in the center between plates are collected in Tables S1,S2. In Fig. S7 we showed the change in the average number of HB, HB-donors and HB-acceptors for the stable hydration states and the intermediate regions (configurations **a-f** on $D_{\mathrm{H_2O}} = f(\Delta l)$ profile - see **main report, Fig. 3**).

Nature of forces acting across clay plate

In Fig. S8 we showed the relevant distances between water molecules separated by the clay plates (illustrated for plates separated by 9.8 Å). The separation between water molecules across plates varies in the range 9.6-14.6 Å (including only the first water shell external to clay plates).

In Fig. S9, the oxygen-oxygen interactions are shown as a function of distance. Although coulombic interactions due to the hydrogen atoms are neglected, it is clear that electrostatics dominates the interactions.

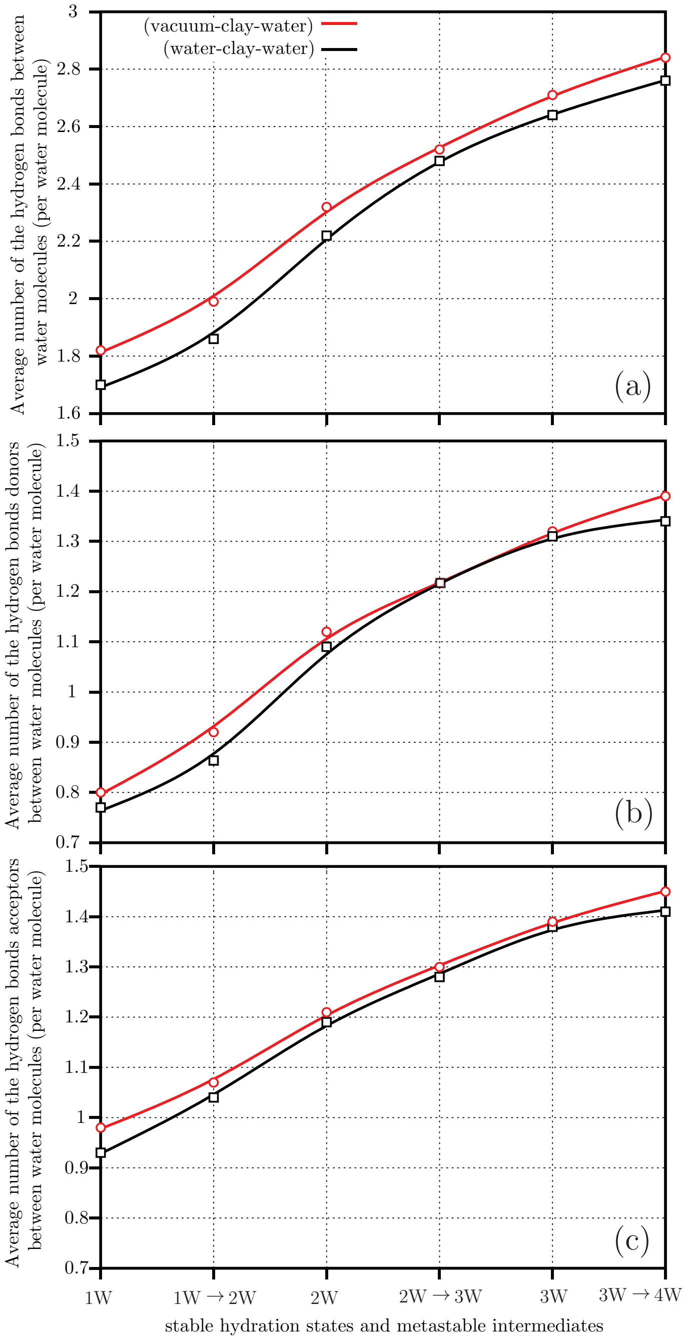

Figure S7: Evolution of the average number of hydrogen bonds (a), hydrogen bond donors (b) and acceptors (c) per water molecules. The hydrogen bonds are analyzed for the interlayered water molecules using the geometric criteria.

Magnitude of collective water-water coupling across clay-plates

In order to understand the nature of water-water coupling across clay plates, we decompose the interaction energies into components arising from water-water, clay-water, clay-clay interactions (for the relevant atomic subsystem - Fig. S10).

The configuration energy is a sum of coulombic and short-range (van der Waals) contributions (see Fig. S10). Here, we estimated the magnitude of water-water coupling across clay plates as:

$$\Delta E = E_1 - (E_2 - E_3) - (E_4 - E_3) - E_3 = E_1 + E_3 - E_2 - E_4 \tag{S2}$$

where E_i is the energy term (van der Waals, coulobmic) describing interactions between all atoms in a given subset (see Fig. S10 b-e). Our coupling energy decomposition method is illustrated for a few configurations in Table S3. The

Table S2: Hydrogen bonding statistics in the center between clay plates for the key clay-separations corresponding to the fully developed water layers (1W , 2W, 3W) and intermediate spacing corresponding to the oxygen density peak splitting/restricted water mobility (1W→2W, 2W→3W, 3W→4W).

Configuration (see Fig. 2 in the manuscript)	Average number of hydrogen bonds $\langle N_{\mathrm{H-bonds}} \rangle_{center}$	Average number of H-bond donors $\langle N_{\mathrm{H-bonds(donors)}} \rangle_{center}$	Average number of H-bond acceptors $\langle N_{\mathrm{H-bonds(acceptors)}} \rangle_{center}$
water only between the clay-plates; vacuum outside			
1W	2.73	1.37	1.35
1W→2W (1-2W)	3.20	1.68	1.52
2W	3.42	1.75	1.67
2W→3W (2-3W)	3.66	1.83	1.84
3W	3.55	1.76	1.79
3W→4W (3-4W)	3.61	1.79	1.82
water between and outside the clay-plates			
1W	2.73	1.37	1.36
1W→2W (1-2W)	3.16	1.65	1.52
2W	3.42	1.75	1.67
2W→3W (2-3W)	3.66	1.83	1.83
3W	3.56	1.77	1.79
3W→4W (3-4W)	3.61	1.79	1.82

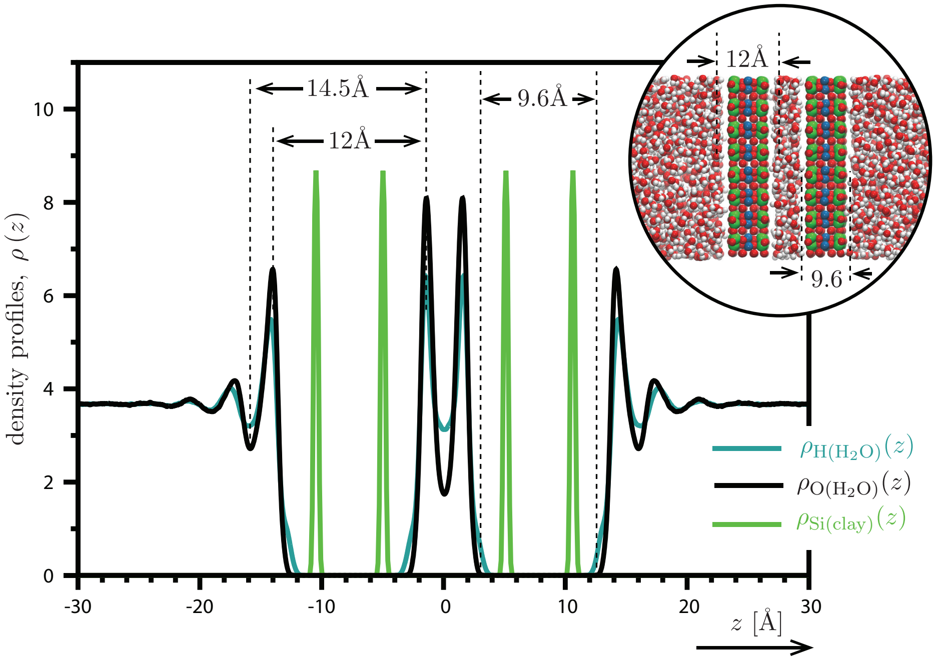

Figure S8: The water-water (oxygen-oxygen) separation across clay plates: minimal separation equals 9.6 Å, the average separation (peak-to-peak) equals 12 Å, whereas interlayer water - first external solvation shell equals 14.5Å. (Illustrated for clay-plates separated by $\Delta l = 9.8$ Å).

interactions between water ensembles across clay plates are dominated by the electrostatics (Table S3).

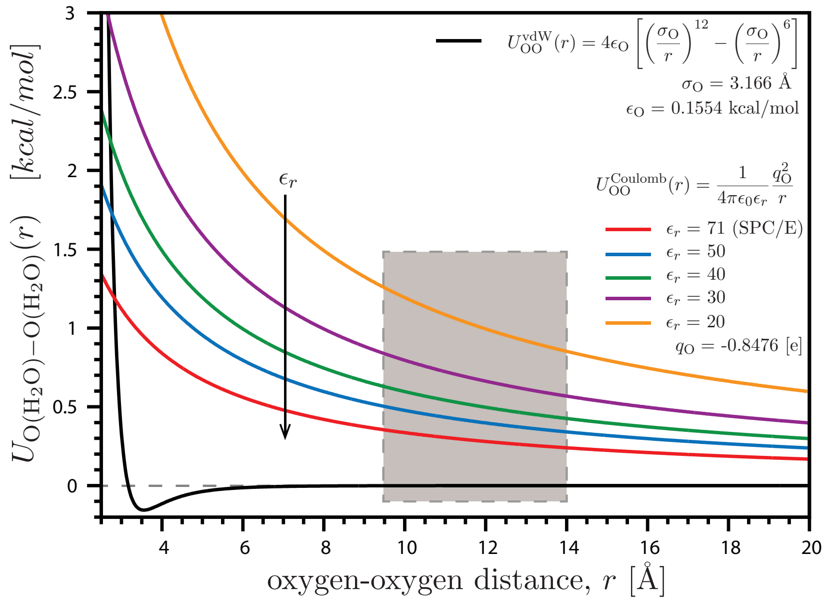

Figure S9: Oxygen-oxygen interactions as function of a atomic separation. The van der Waals interactions are described using Lennard-Jones potential (ϵ, σ values as in the SPC/E water model). The screening of the electrostatic interactions are included by ϵ_r. In the fully atomistic molecular modeling reported in this paper ϵ_r equals 1. The oxygen-oxygen separation that is relevant for the water-water coupling across clay plate are indicated by the brown box.

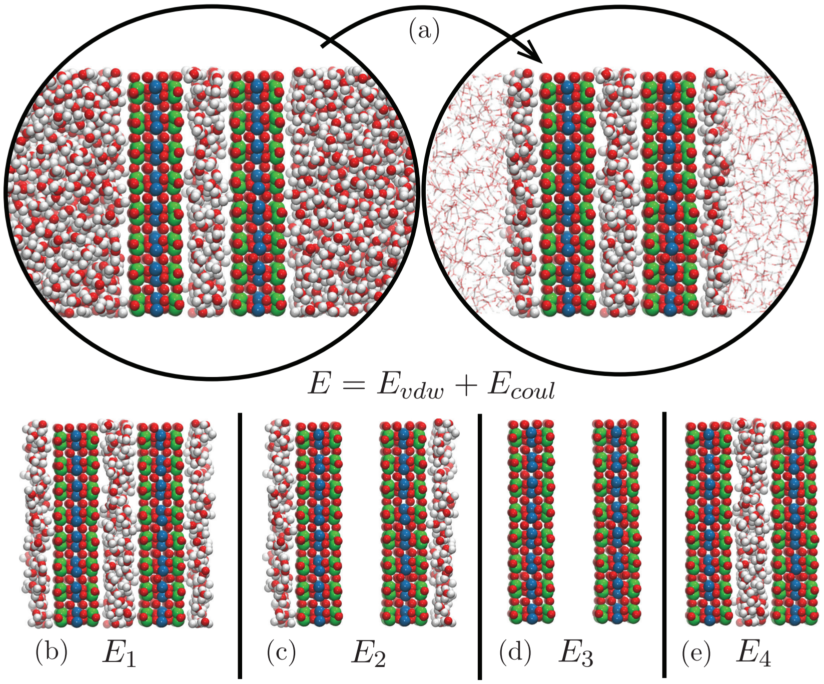

Figure S10: Energy decomposition procedure. The interactions are considered between atoms in the subsystem (a) composed of the clay plates, intercalated water and the first water solvation shells external to the clay plates (E_1, b). In order to understand the collective water-water coupling across clay plates we calculated the interaction energies in a series of atomic subsystems ($E_2 - E_4$, c-e) in which certain components were switched off.

Table S3: Magnitude of the water coupling across clay-plates. The coupling energy, ΔE, is decomposed into short range (van der Waals, ΔE_{vdw}) and long range (electrostatic, ΔE_{coul}) contributions (see Fig. S10). The energies (E) are calculated on the fly (incorporated into Velocity-Verlet integrator); quantities are divided by the number of atoms in considered subset (Fig. S10b).

Configuration (Fig. 2 in the manuscript)	ΔE_{vdw} $[kcal/mol/atoms]$	ΔE_{cou} $[kcal/mol/atoms]$	$\Delta E = \Delta E_{vdw} + \Delta E_{cou}$ $[kcal/mol/atoms]$
2W	0.0025	-186.5381	-186.5356
2W$\rightarrow$3W (1-2W)	-0.0011	-230.6097	-230.6109

References

[1] R Kumar, J R Schmidt, and J L Skinner. Hydrogen Bonding Definitions and Dynamics in Liquid Water. *J. Chem. Phys.*, 126(20):204107–13, 2007.

[2] A Luzar and D Chandler. Hydrogen-Bond Kinetics in Liquid Water. *Nature*, 379(6560):55–57, 1996.

[3] P. Zarzycki. Interfacial Water Screens the Protein-Induced Transmembrane Voltage. *J. Phys. Chem. B*, 119(4):1474–1482, January 2015.
